# Ecological Monitoring and Health Research in Luambe National Park, Zambia: Generation of Baseline Data Layers

**DOI:** 10.1007/s10393-016-1131-y

**Published:** 2016-06-23

**Authors:** Neil E. Anderson, Paul R. Bessell, Joseph Mubanga, Robert Thomas, Mark C. Eisler, Eric M. Fèvre, Susan C. Welburn

**Affiliations:** 1 The Royal (Dick) School of Veterinary Studies and the Roslin Institute, University of Edinburgh, Easter Bush Campus, Roslin, EH25 9RG UK; 2 Department of Veterinary Services, Lusaka, Zambia; 3 Biomedical Sciences, Edinburgh Medical School, College of Medicine and Veterinary Medicine, The University of Edinburgh, Edinburgh, UK; 4 School of Veterinary Sciences and Cabot Institute, University of Bristol, Langford House, Langford, Bristol, BS40 5DU UK; 5 Institute of Infection and Global Health, University of Liverpool, Leahurst Campus, Neston, CH64 7TE UK; 6 International Livestock Research Institute, Old Naivasha Road, PO Box 30709-00100, Nairobi, Kenya; 7 Division of Infection and Pathway Medicine, Biomedical Sciences, Edinburgh Medical School, College of Medicine and Veterinary Medicine, The University of Edinburgh, Chancellor’s Building, 49 Little France Crescent, Edinburgh, EH16 4SB UK

**Keywords:** distance sampling, fuzzy classification, Luangwa Valley, *Phacochoerus africanus*, trypanosome, warthog, wild mammal density, Zambia

## Abstract

Classifying, describing and understanding the natural environment is an important element of studies of human, animal and ecosystem health, and baseline ecological data are commonly lacking in remote environments of the world. Human African trypanosomiasis is an important constraint on human well-being in sub-Saharan Africa, and spillover transmission occurs from the reservoir community of wild mammals. Here we use robust and repeatable methodology to generate baseline datasets on vegetation and mammal density to investigate the ecology of warthogs (*Phacochoerus africanus*) in the remote Luambe National Park in Zambia, in order to further our understanding of their interactions with tsetse (*Glossina* spp.) vectors of trypanosomiasis. Fuzzy set theory is used to produce an accurate landcover classification, and distance sampling techniques are applied to obtain species and habitat level density estimates for the most abundant wild mammals. The density of warthog burrows is also estimated and their spatial distribution mapped. The datasets generated provide an accurate baseline to further ecological and epidemiological understanding of disease systems such as trypanosomiasis. This study provides a reliable framework for ecological monitoring of wild mammal densities and vegetation composition in remote, relatively inaccessible environments.

## Introduction

Understanding the structure of natural ecosystems forms the basis for understanding the processes within those ecosystems, including the transmission of infectious and vector-borne diseases. Remotely sensed datasets and geographical information systems (GIS) have been widely used to further our understanding of these systems. This technology has not only helped in the study of the global drivers of ecological change, but is also invaluable for understanding the biotic and abiotic factors influencing ecosystems at much smaller scales. GIS technology has become an integral component of many conservation programmes and the development of trans-disciplinary approaches such as Conservation Medicine, One Health and EcoHealth have highlighted its utility. However, recent moves to adopt ecosystem-based approaches within conservation and development programmes have highlighted frequent deficiencies in baseline ecological data, particularly in developing countries (Rapport et al. 1998).

For an area with such an internationally acclaimed biodiversity, relatively little ecological data exist for the Luangwa Valley in eastern Zambia (latitude −10.4° to −15.6° and longitude 30.2° to 33.1°). Although many valuable mapping and vegetation studies have been conducted, they either lack detail (Trapnell 1950; Naylor et al. 1973; Phiri 1989; Marks 2005) or have restricted geographical coverage (Astle et al. 1969; Smith 1998; Yang and Prince 2000). Similarly, published faunal surveys for the Luangwa Valley are rare and no peer-reviewed published data are available for many areas. Studies have been conducted in game management areas (GMAs) surrounding some of the national parks (Ndhlovu and Balakrishnan 1991; Lewis et al. 2011), and many of the species recorded historically in the mid-Luangwa Valley have been documented (Astle 1999). Aerial surveys have been conducted in the core parts of the Luangwa Valley on behalf of the Zambian Wildlife Authority (ZAWA) (Simukonda 2011) and as part of the Community Markets for Conservation Programme (COMACO) (Olive et al. 2012; Frederick 2013). The population of hippopotamus (*Hippopotamus amphibious*) has recently been surveyed and extensively studied (Wilbroad and Milanzi 2010; Chansa et al. 2011a; Chansa et al. 2011b). However, there is a clear need for more high-resolution data to enable active monitoring of ecosystem health in the valley.

There has been much interest in the role of warthogs (*Phacochoerus africanus*) as natural reservoir hosts for African swine fever (Plowright et al. 1969; Wilkinson et al. 1988), trypanosomiasis (Dillmann and Townsend 1979; Claxton et al. 1992) and bovine tuberculosis (Bengis et al. 2002; Michel et al. 2006). Warthog burrows not only provide a refuge for warthogs from predators and extremes of temperature, but they also provide a refuge for many parasites (Cumming 1975; Somers et al. 1994). The cool shady conditions in the entrance to warthog burrows provide an ideal refuge for tsetse flies during the heat of the day (Pilson and Pilson 1967), and the burrows are important sites for larviposition by female flies (Leak 1998). Warthogs are also a preferred host for *Glossina morsitans* species of tsetse flies, and a close ecological association between tsetse and warthog has been proposed (Pilson and Pilson 1967; Torr 1994; Leak 1998). A study of tsetse ecology in Luambe National Park (LNP) revealed that *Combretum-Terminalia* vegetation supports the highest apparent density of *G. m. morsitans* and thicket the highest apparent density of *G. pallidipes* (Anderson 2009). Warthogs have been shown to carry a moderate prevalence of trypanosomes and the human-infective *Trypanosoma brucei rhodesiense*, the cause of human African trypanosomiasis (HAT), has been identified in warthogs in the Luangwa Valley (Dillmann and Townsend 1979; Anderson et al. 2011). As a wide variety of other hosts are fed on by tsetse to varying degrees (Clausen et al. 1998) and are components of the natural reservoir community for trypanosomiasis in the Luangwa Valley (Anderson et al. 2011), it is important to understand more about the density and distribution of wild animal hosts within these ecosystems.

The majority of investigations into trypanosomiasis in wildlife have focussed on estimation of the prevalence of infection. Historically, prevalence was largely interpreted in terms of host susceptibility to infection and, to a lesser extent, host preference by tsetse. However, the importance of ecological and behavioural factors in the transmission of wildlife disease is now recognised (Cross et al. 2009). Factors such as habitat preference, resource use, territoriality, group size and group density contribute to a complex social and spatial structure for wildlife disease. Understanding the structure and distribution of both plant and animal communities is therefore critical for clarifying the nature of contact between hosts and vectors, and its impact on disease transmission. In a detailed review of the ecological factors influencing the epidemiology of trypanosomiasis in the Luangwa and Zambezi Valley ecosystems, Munang’andu et al. (2012) identified host distribution and abundance as having a significant influence on the survival of tsetse and therefore on trypanosomiasis epidemiology. Many other factors are also important including daily activity patterns of hosts and seasonal migration behaviour. Tsetse distribution and abundance is largely driven by climatic factors, host abundance and vegetation (Robinson et al. 1997). A better understanding of the distribution and characteristics of both mammal and plant communities is therefore likely to improve our management of HAT.

Here we generate accurate high-resolution datasets of vegetation and large mammal density in the remote, relatively inaccessible LNP within the Luangwa Valley, in order to investigate the ecology of warthogs and to further our understanding of their interactions with *Glossina* spp., vectors of trypanosomiasis.

## Methods

### Study Area

**Figure 1:**
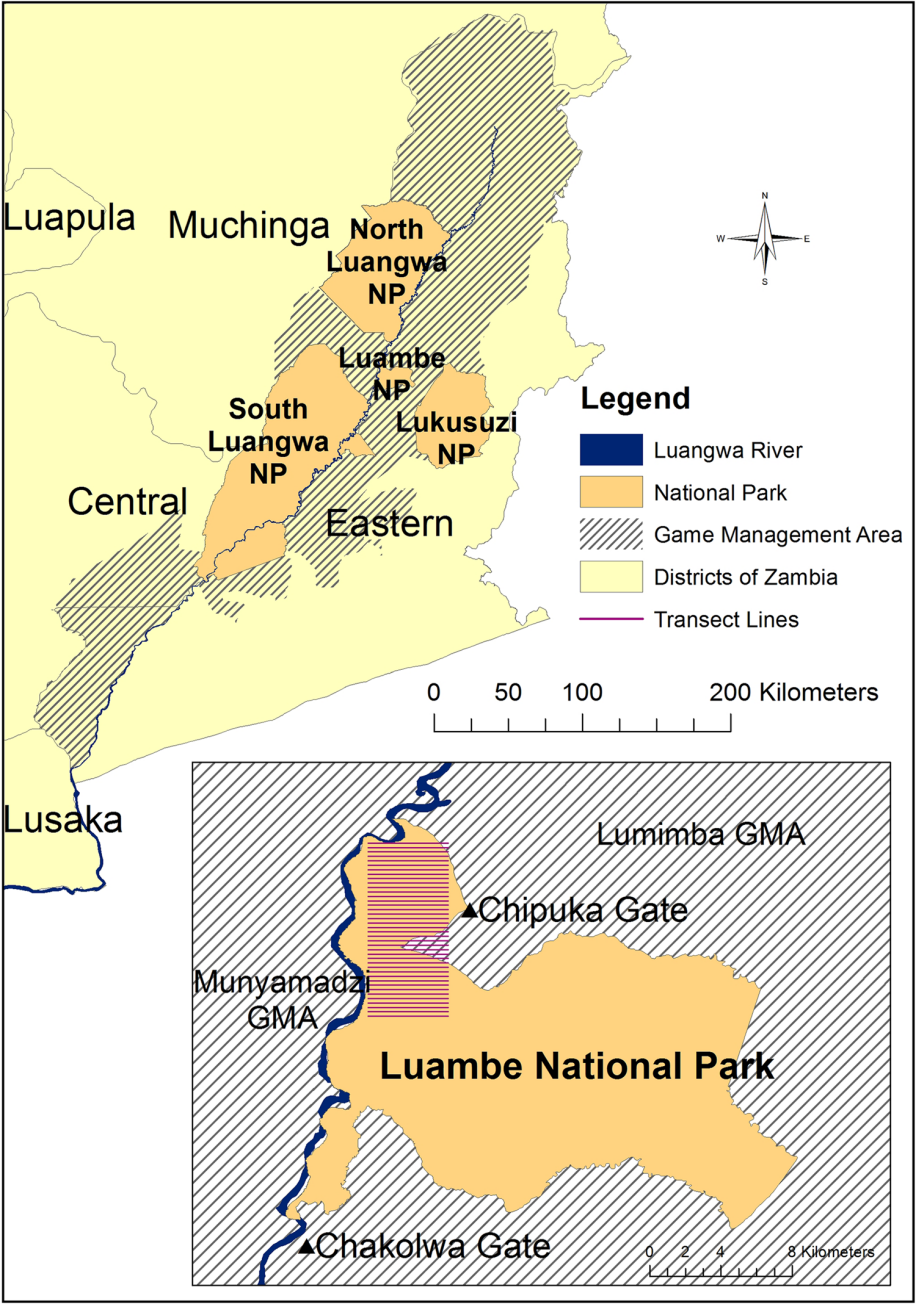
Map of the Luangwa Valley, with inset map showing the location of ground transects within Luambe National Park.

The Luangwa Valley lies in Muchinga, Eastern and Central Provinces of Zambia, forming an extension of the Great Rift Valley. LNP is a relatively small national park in the mid-Luangwa Valley, situated between the larger North and South Luangwa national parks on the other side of the Luangwa River (Fig. 1). It is poorly developed with minimal infrastructure, few roads and little accessibility during the rains (December to March). It is situated close to the historical sleeping sickness nidus in Nabwalia (Kinghorn et al. 1913).

### Landcover Classification

#### Landsat 7 ETM+ Data

Landsat 7 ETM+ data with a spatial resolution of 30 m were selected for this study. The most recent L1G Landsat satellite image covering the study area was downloaded from the Global Land Cover Facility maintained by the University of Maryland (image acquisition date 04/10/2001, path 170, row 069, cloud cover 0%) (NASA Landsat Programme 2004). Proprietary satellite image processing software Erdas Imagine 8.4 (Leica Geosystems AG, Atlanta, USA) was used for all image processing and classification procedures.

#### Classification Scheme

The classification scheme selected is, to a degree, dictated by the objectives of the study in question (Congalton 1991). In this case, an important objective was to produce a dataset suitable for use as a GIS base layer for the design and spatial analysis of warthog and tsetse surveys planned for the park. Therefore, the classification level needed to distinguish between woodland classes, for example, rather than to simply classify to the broad physiognomic vegetation unit level (i.e. woodland).^1^ The classification of vegetation at the physiognomic level by White (1983) was first used to understand the vegetation units represented in the park. These physiognomic units were then further divided into individual land cover classes (Table 1). Particular reference was made to the previous detailed studies of the vegetation by Astle et al. (1969) and Smith (1998) with allowances made for local differences in vegetation type found in LNP.

**Table 1:** Vegetation Classes by Physiognomic Unit.

Physiognomic vegetation unit	Vegetation class	Characteristic tree/shrub species	Acronym	Abbreviation
Woodland	Acacia woodland	*Acacia kirkii*	None	AW
	*Combretum-Terminalia* woodland	*Combretum* species (*C. fragrans*, *C. collinum, C. imberbe*) *Terminalia sericea*	*Combretum* woodland	CTW
	Mopane woodland	*Colophospermum mopane*	None	MW
	Riverine woodland and thicket	*Diospyros mespiliformis*, *Kigelia africana, Trichilia emetica, Afzelia quanzensis*, *Combretum obovatum*	Riverine woodland	RWT
Scrub woodland	Hill scrub miombo woodland	*Julbernardia globiflora*	Scrub miombo woodland	HSMW
	Mopane scrub woodland	*Colophospermum* *mopane*	Mopane scrub	MSW
Thicket	Thicket	*Schrebera trichoclada*, *Diospyros quiloensis, Combretum* species (*Combretum obovatum*)	None	TH
Grassland	Grassland	Occasional *Combretum obovatum, Kigelia africana,* and *Colophospermum mopane*	None	G
	Semi-permanent water/aquatic-association grassland	*Combretum imberbe*	Aquatic grassland	SPW/AAG

#### Ground-Truthing

Ground-truthing was conducted to confirm the classification scheme and collect reference training and test data in August and September, 2005. Evaluation of vegetation class relied on qualitative observations of vegetation physiognomy and predominant tree or shrub species present. Tree and plant species were identified with assistance from standard field guides covering the southern African region (Van Wyk and Van Wyk 1997; Coates Palgrave 2003a) and the Luangwa Valley (Smith 1995; Coates Palgrave 2003b). Spatial area was used to create a sample frame for collection of reference data, with polygons of homogenous vegetation as the sample unit. Twenty or more sample units were collected for all classes using a hand-held global positioning system (GPS), except for acacia woodland (sixteen) and hill scrub miombo woodland (nine) whose limited distribution precluded the collection of more data. For practical reasons, reference data for the water class was created from the non-classified Landsat image.

#### Classification

As collection of truly homogeneous polygons of vegetation was difficult due to the occurrence of natural mosaics, a classification approach based on fuzzy set theory was used (Wang 1990; Foody 1996). Fuzzy set theory allows for degrees of truth to be represented in algorithms allowing for joint membership of sets, or fuzzy boundaries. In image classification, it may be used to replace conventional probability theory in the classification process to create a fuzzy partition of the spectral space. This allows joint membership of classes by pixels represented by membership grades, the important feature being that mixed pixels are represented in the output classified image.

Reference polygons were firstly converted into regularly spaced points at 30-m intervals (each point effectively representing the value of one pixel) as the accuracy assessment algorithm required point data rather than polygon data. A subset of 25% of the points in each class were randomly selected and withheld from the training data, to be used as the test data. The training data were then used to create spectral signatures for each vegetation class. As the reflectance and emittance properties vary for different vegetation types, a spectral response pattern referred to as a spectral signature may be produced for each class from the training data (Lillesand et al. 2004). The image was classified using a maximum likelihood classifier with the additional activation of the fuzzy classification function (Pouncey et al. 1999). The feature space non-parametric decision rule was applied first and pixels in areas of overlap using this algorithm were then classified using the maximum likelihood parametric decision rule. Any unclassified pixels using the non-parametric decision rule were also classified using the parametric maximum likelihood rule. The option to select eight best classes was chosen resulting in an eight-layered fuzzy image. This was then processed into one layer to produce an output land cover map using the fuzzy convolution facility (Pouncey et al. 1999). A 3 × 3 window size was selected and the neighbour weighting option used with a neighbourhood weight factor of 0.5. All eight layers were used to perform the operation.

#### Image Evaluation

Classified images were examined by direct visualisation of the output image and graphical evaluation of spectral response patterns. Transformed divergence values were calculated to assess signature separability. An error matrix was generated to compare test and classified data; the overall accuracy, overall kappa statistic and modified estimator of kappa for stratified random sampling were calculated (Stehman 1997). Class level producer’s and user’s accuracies were calculated along with the conditional kappa values. The accuracy of the classification produced by the fuzzy algorithm was compared to a standard maximum likelihood classification using McNemar’s test for significance with the continuity correction (Foody 2004).

### Distance Sampling Survey

#### Transect Design

The newly created land cover dataset was used to design a mammal density survey using distance sampling methods. The primary objective was to estimate the abundance of warthog and other mammalian hosts of trypanosomes and the secondary objective to map warthog burrow distribution. A ground transect survey was used rather than aerial survey techniques to enable more accurate estimation of the density of smaller species which are important hosts for trypanosomiasis. The study was conducted during the dry season from August to October, 2006, in the north of the park (the location for a concurrent tsetse survey). A single random starting point was generated to create a systematic grid of 40 parallel transects perpendicular to the Luangwa River. The length of each transect was 4.5 km and the distance between parallel transects was 250 m, providing a total survey length of 180 km (inset map, Fig. 1).

#### Survey Protocol

The same personnel were used throughout the survey, one being an observer and one a measurer and recorder. Transects were conducted on foot using a hand-held GPS and all observations of mammal species and warthog burrows were recorded. The perpendicular distance from the transect line was measured using a laser range-finder to ensure accuracy. All transects were started at approximately 6:30 am, the peak activity period for the majority of species of interest, to ensure consistency across transects. One transect line would be walked in an easterly direction and the personnel would continue 250 m beyond the finish before walking one kilometre either south or north to return along a different transect line in a westerly direction. This protocol was followed in order to reduce the undesirable effects caused by evasive movement of animals following disturbance during the preceding transect.

#### Data Analysis

The conventional distance sampling engine packaged within the specialist distance sampling software program Distance was used for analysis (Thomas et al. 2006). The process recommended by Buckland et al (2001) was followed with the transect lines defined as the sampling units. A series of plausible models combined with expansion terms were fitted to the data. A maximum of five adjustment terms were fitted using AIC by the sequential method. Histograms and qq plots were examined to assess data and model fit. In all cases, data were grouped into distance intervals, and truncation was carried out to remove outliers. The size-bias regression method was used to adjust for detection bias for clusters of animals (Buckland et al. 2001). Exact distance values, rather than distance intervals, were used in size-bias regression calculations. The non-parametric bootstrap method was used to estimate variance with resampling of 999 samples, seeded from the system clock. Confidence intervals were calculated as 2.5% and 97.5% quantiles of the bootstrap estimates. Final models were selected on the basis of the AIC, variance and chi-squared goodness of fit.

The wild mammal survey was analysed with stratification by species and habitat (vegetation class). For stratification by species, the observations for all species with a sample number of 40 or greater were examined using the detection function specific for that species. For species with an inadequate sample number for this approach, the global detection function was used. For the habitat study, the vegetation class for each observation was extracted from the classified image and density estimates made using the stratum specific detection function if the sample number was adequate. Where numbers of observations were not sufficient, data were pooled with the most similar habitat type and estimates made using the global detection function for the pooled data. Observations of rodents were not included in the analysis as they could not be identified accurately to a species level from a distance. Separate density estimates were made for warthog burrows in use at the time of the survey, as well as for the total number of warthog burrows detected (including inactive burrows).

## Results

### Land Cover Classification

**Figure 2:**
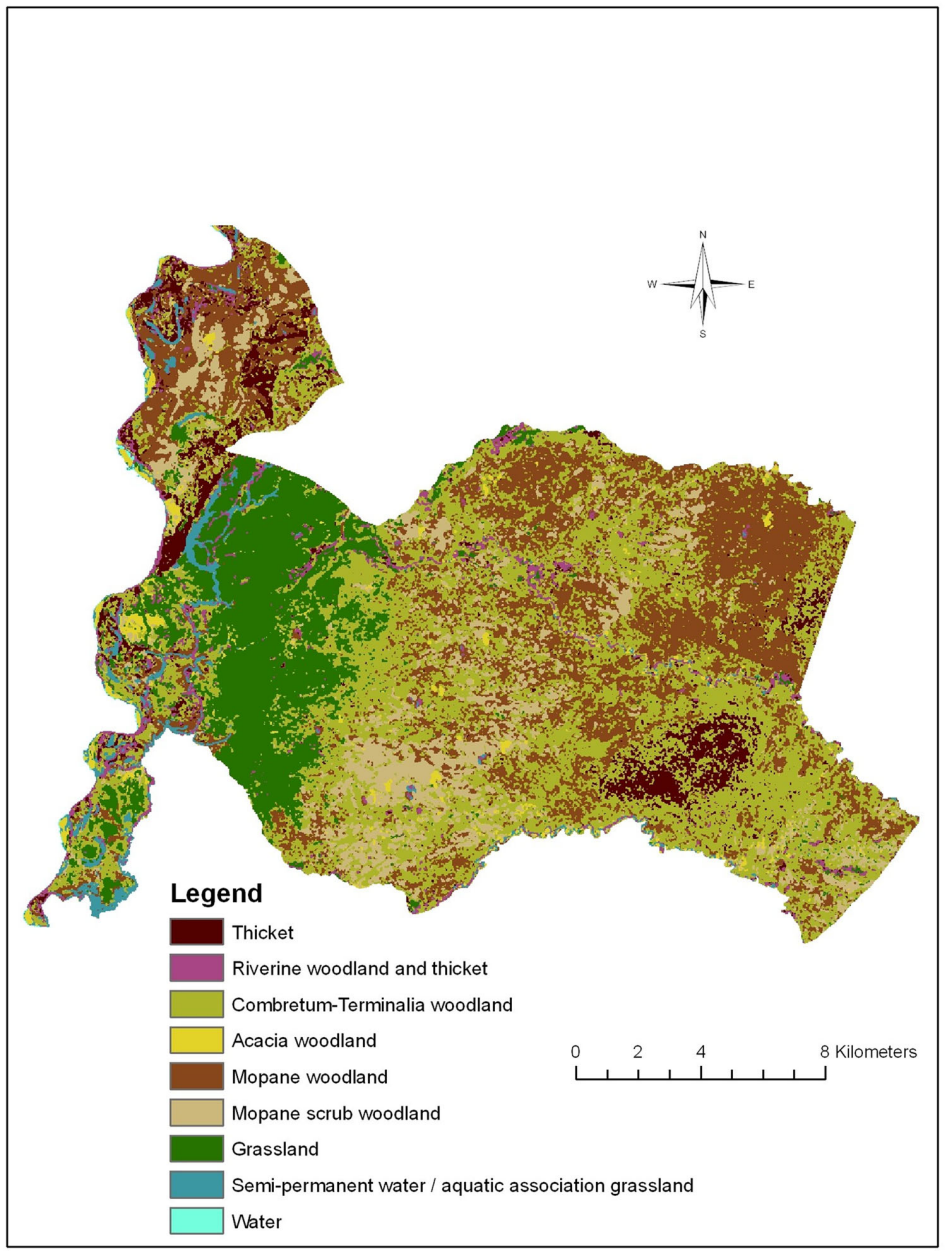
Land cover classification of Luambe National Park.

Ten land cover classes were identified during the ground-truthing study (Table 1). The hill scrub miombo class was very small and could not be accurately mapped so was removed from the final classification (see discussion). The overall accuracy of the classification was 71.2% (95% CI 65.3–76.7%). The image produced by the fuzzy classifier was significantly more accurate than that produced using a standard maximum likelihood classifier (McNemar’s chi-squared = 4.6875, df = 1, *P* value = 0.030). The error matrix is presented with the conditional kappa for the classified data rather than the reference data (Table 2). The area of the park as calculated from the classified image was 331 km^2^ (33119 hectares) and the total perimeter length was 142 km (142102 m). The complete dataset for the final classified image (Fig. 2) is available for download via the ShareGeo open access repository at http://hdl.handle.net/10672/606.

**Table 2:** Error Matrix for the Classification.

Class name	Reference data										
	MSW	TH	RWT	CTW	G	SPW/AAG	AW	W	MW	User’s accuracy	95% CI
Classified data											
MSW	**17**	2	0	1	5	2	1	0	0	61	41–78
TH	0	**18**	10	1	0	1	0	0	2	56	38–74
RWT	0	0	**14**	1	1	1	0	0	0	82	57–96
CTW	3	5	5	**22**	4	2	3	1	2	47	32–62
G	0	0	0	0	**50**	1	3	0	0	93	82–98
SPW/AAG	0	1	0	0	0	**9**	0	0	0	90	55–100
AW	0	0	0	0	0	0	**7**	0	0	100	59–100
W	0	0	0	0	0	0	0	**18**	0	100	81–100
MW	7	0	3	0	2	2	2	0	**28**	64	48–78
Producer’s accuracy	63	69	44	88	81	50	44	95	88		
95% CI	42–81	48–86	26–62	69–97	69–90	26–74	20–70	74–100	71–96		
Conditional kappa	0.56	0.5	0.81	0.35	0.91	0.9	1	1	0.56		

Values in bold are correctly classified pixels. The sum of these values divided by the total number of samples in the matrix provides the overall accuracy

Overall accuracy = 71.2% (95% CI 65.3–76.7%).

Overall kappa statistic (*κ*) = 0.67.

Estimator of kappa (KS) for stratified random sampling (Stehman 1997) = 0.74.

### Mammal Density

Details of observations, cluster sizes and density estimates for all species recorded during the survey are presented in Table 3. The densities of species with 40 or more observations should be considered more reliable than the other estimates as they were estimated using the stratum detection function. The overall density estimate of wild mammals in the study area, excluding rodents, was 17.32 animals/km^2^ (95% CI 12.69–24.59). An additional 315 rodents were observed, including 249 mopane squirrels (*Paraxerus cepapi*). Observations of warthogs were most frequently made in aquatic grassland and *Combretum* woodland with nearly a quarter of all observations made in each of these classes.

**Table 3:** Animals Observed During the Transect Survey and Density Estimates for the More Abundant Species.

Common name	Scientific name	Observations	Mean cluster size	Truncation distance (m)/intervals	Observations (post-truncation)	Detection function	AIC	ESW (m)	Estimated cluster size (95% CI)	Density (95% CI)
Common warthog	*Phacochoerus africanus*	46	2.3	230/6	38	Stratum	98.04	81	2.41 (1.88–2.85)	3.14 (1.93–5.98)
Impala	*Aepyceros melampus*	150	5.3	200/7	123	Stratum	436.27	108	4.26 (3.32–5.57)	13.45 (8.75–23.47)
Puku	*Kobus vardonii*	216	5.9	500/9	206	Stratum	753.35	174	6.33 (4.76–8.13)	20.85 (12.38–35.78)
Baboon	*Papio cynocephalus*	34	13.6	380/10	33	Global	1735.34	106	17.69 (10.28–28.02)	15.37 (7.48–26.16)
Bushbuck	*Tragelaphus scriptus*	12	1.3	380/10	12	Global	1735.34	106	1.21 (1.00–1.68)	0.38 (0.18–0.68)
Elephant	*Loxodonta africana*	20	3.6	380/10	16	Global	1735.34	106	5.50 (2.67–9.28)	2.32 (0.88–4.68)
Sharpe’s grysbok	*Raphicerus sharpei*	11	1.1	380/10	11	Global	1735.34	106	1.07 (1.00–1.28)	0.31 (0.15–0.55)
Banded mongoose	*Mungos mungo*	5	3.2	–	–	–	–	–	–	–
Burchell’s zebra	*Equus burchellii*	12	7.8	–	–	–	–	–	–	–
Common waterbuck	*Kobus ellipsiprymnus*	6	2.2	–	–	–	–	–	–	–
Cookson’s wildebeest	*Connochaetes taurinus* cooksoni	4	8.8	–	–	–	–	–	–	–
Greater kudu	*Tragelaphus strepsiceros*	3	4.3	–	–	–	–	–	–	–
Serval	*Leptailurus serval*	1	1.0	–	–	–	–	–	–	–
Slender mongoose	*Galerella sanguinea*	2	1.0	–	–	–	–	–	–	–
Southern reedbuck	*Redunca arundinum*	7	2.9	–	–	–	–	–	–	–
Spotted hyaena	*Crocuta crocuta*	1	2.0	–	–	–	–	–	–	–
Vervet monkey	*Cercopithecus aethiops*	5	3.4	–	–	–	–	–	–	–
Total	–	784	3.9	–	–	–	–	–	–	–

Models with a half-normal key function and cosine series expansion provided the best fit for all species. Density is number per square kilometre.

Large mammal density aggregate estimates by vegetation class are presented in Table 4. Observations for the riverine woodland class and thicket class were pooled in order to enable a class level density estimate to be made using the global detection function of the two classes. *Combretum* woodland and mopane scrub were similarly combined to obtain a class level estimate. Few observations were recorded for the acacia woodland and mopane scrub classes meaning that no estimate was possible for the former, and a less reliable estimate was possible for the latter, compared with other classes.

**Table 4:** Summary of the Estimated Densities for Large Mammals^a^ by Vegetation Class in the Study Area.

Habitat	Truncation distance (m)/intervals	Observations (post-truncation)	Detection function	AIC	ESW (m)	Estimated cluster size (95% CI)	Density (95% CI)
Aquatic grassland	430/9	73	Stratum	284.96	201.71	4.19 (2.66–5.84)	7.14 (4.09–12.41)
*Combretum* woodland	165/8	43	Global	230.9	62.48	2.60 (2.00–3.80)	6.45 (3.49–10.01)
Grassland	400/15	178	Stratum	947.36	190.79	7.23 (4.93–10.25)	41.04 (23.12–68.20)
Mopane scrub	165/8	21	Global	230.9	62.48	3.18 (1.41–6.60)	3.84 (1.22–8.42)
Mopane woodland	210/9	93	Stratum	344.27	86.04	5.64 (3.86–7.48)	25.62 (11.58–58.17)
Riverine woodland	380/10	38	Global	248.42	85.341	8.12 (4.17–15.36)	12.13 (5.51–25.57)
Thicket	380/10	39	Global	248.42	85.341	3.00 (1.77–4.43)	5.33 (2.96–8.82)
Total	380/10	489	Global	1864.63	109.8	5.09 (4.10–6.24)	17.32 (12.69–24.59)

Models with a half-normal key function and cosine series expansion term provided the best fit to the data when stratified by vegetation type and hazard rate key function with simple polynomial expansion term when not stratified.

^a^Baboon, Bushbuck, Elephant, Sharpe’s Grysbok, Impala, Greater Kudu, Puku, Southern Reedbuck, Serval, Spotted Hyaena, Vervet Monkey, Warthog, Common Waterbuck, Cookson’s Wildebeest and Burchell’s Zebra

### Warthog Burrow Density and Distribution

**Figure 3:**
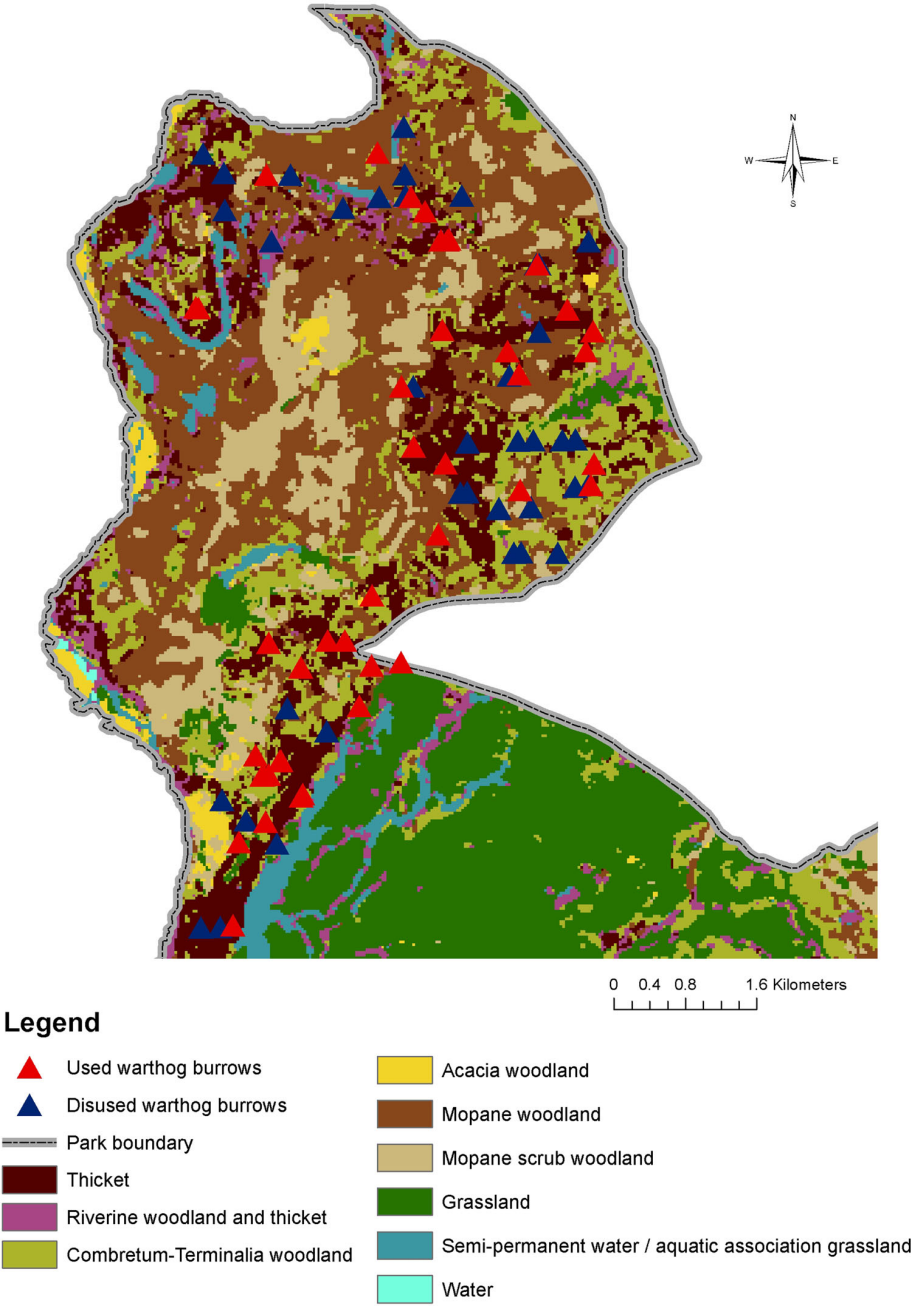
Geographical distribution of warthog burrows in the study area.

A total of 86 warthog burrows were detected during the transect survey, 42 of which appeared to have been recently used. The number of observations detected permitted an overall estimate of density, but was not sufficient to allow density estimates stratified by habitat (Table 5). The spatial distribution of warthog burrows is presented in Figure 3. The vast majority were observed in or around a slightly elevated band of *Combretum* woodland and thicket surrounding a large central area of mopane woodland and mopane scrub.

**Table 5:** Density Estimates for Warthog Burrows in the Study Area.

Status	Truncation distance (m)/intervals	Observations (post-truncation)	Detection function	AIC	ESW (m)	Density (95% CI)
All burrows	29/7	83	Stratum	242	10.6	21.80 (15.75–37.89)
Used burrows	29/8	40	Stratum	133.26	11.36	9.81 (5.92–14.71)

A model using a half-normal key with a cosine expansion term provided the best fit for both models.

## Discussion

### Performance of the Classification

The overall accuracy of 71.2% for the final classified image was considered to be good and the fuzzy logic algorithm presented statistically significant improvements over the conventional maximum likelihood algorithm. The presence of mixed pixels in the image is likely to account for much of the difference in performance between the two. Mixed pixels have been identified as a major source of error in traditional ‘hard’ classifications that assign only one class to each pixel (Wang 1990; Foody 1996; Benz et al. 2004) and as the most important cause of misclassifications (Foody 2002). Detailed information on joint membership by other classes, particularly around boundary areas, is lost. There is no doubt the training data will have contained some mixed pixels as vegetation exists as a continuum in LNP (as in most natural ecosystems) and classes overlap. Indeed, in his detailed floristic study of North Luangwa National Park, Smith (1998) grouped his vegetation categories into mosaics of vegetation types that could not be mapped separately at his chosen resolution. Although attempts were made to collect homogenous reference data in this study, it will have contained some heterogeneity and represented, in effect, ‘fuzzy’ ground-truth data. An important feature of fuzzy classifiers is that homogenous reference data are not needed.

Most of the classes within the classification scheme performed well, with the exception of the hill scrub miombo class. A small area of this class was identified during the ground survey on the hills in the south eastern section of the park, but only at an altitude of 660 m or greater. The highest point in LNP, based on the 1:250,000 topographical maps (Surveyor-General 1972), is 680 m meaning this class was only present over a very restricted area. As it was exerting a deleterious effect on the accuracy of the rest of the classification, it was removed. Most of this area is mapped by the classification as *Combretum* woodland with some scrub mopane woodland. In reality, it is likely to represent more of a transition zone from *Combretum* woodland to scrub miombo woodland rather than just the latter.

### Vegetation Composition of the Park

As discussed earlier, much of the vegetation of LNP exists in a natural mosaic of vegetation types. However, at a larger scale, several classes occur in fairly discreet zones, notably mopane woodland, mopane scrub and grassland. The two forms of *Colophospermum mopane* vegetation together are dominant over large areas of the park covering 37% of the total area. The large grassland habitats formed by the floodplains of the Luangwa River tributaries are a significant component of the park covering 14%. Thicket vegetation also forms fairly clear zones in places, but interdigitates with *Combretum* woodland in others. Aquatic grasslands, in the form of permanent or semi-permanent lagoons, account for a much smaller proportion of the total park area, but are a very characteristic feature of LNP. Riverine woodland mainly flanks the Luangwa River, but is also found in patches by the larger tributaries and lagoons. Detailed descriptions of the vegetation classes in this study may be found elsewhere (Anderson 2009).

### Species Densities

Despite its small size, LNP has some distinctive wildlife habitats and supports populations of several globally threatened or endemic mammal species. The wildlife density estimates presented here represent the most detailed published information to date and can act as a baseline for on-going research and monitoring. Overall mammal densities were relatively low with some notable exceptions such as puku (*Kobus vardonii*).

Of the most abundant species, only warthog are known to be preferred hosts for tsetse (Clausen et al. 1998). Warthogs are generally a successful species and density estimates vary from 15 km^−2^ (Cumming 1975) to 30 km^−2^ (Estes 1993) in the best habitat (fertile alluvial soils), and less than 1 km^−2^ (Cumming 1975) in less favourable areas. Densities are highest in short grassland or wooded grassland areas (Rodgers 1984) and mosaics of suitable wet and dry season habitat are important (Cumming 1975). The density of warthog estimated in this study (3.14 km^−2^, 95% CI 1.93–5.98) was comparable with the density recorded in nearby Upper Lupande GMA (2.2 km^−2^) and the Zambezi Valley, Zimbabwe, but towards the lower end of reported densities (Cumming 1975; Rodgers 1984; Ndhlovu and Balakrishnan 1991). The relatively high proportion of warthog clusters observed in aquatic grassland is notable as it covered only 4% of the transect area. Aquatic grassland presents a reliable dry season source of forage for warthogs which were frequently observed digging for rhizomes of grasses and sedges in this habitat. Observations were comparatively frequent in the *Combretum* woodland habitat, which has a well-developed grass layer. Also notable were high densities of warthog in areas with new grass appearing after a bush fire. Marked local increases in warthog density after dry season fires have been noted before (Cumming 1975). Uncontrolled burning is a regular occurrence in LNP and is also likely to exert a significant selection pressure on the vegetation, especially given the resistant nature of *Combretum* species in particular to fire (Smith 1998). In turn it is likely to have significant effects on the diversity and abundance of fauna.

### Warthog Burrow Distribution

Mapping of the warthog burrows over the classified dataset allowed the spatial pattern and habitat preference for burrow location to be examined. The clear pattern revealed in Figure 3 may be explained by the drainage of the soils, the ease of excavation and the provision of cover from predation. *Combretum* woodland and thicket are generally found on more sandy soils with better drainage in the rains and easier excavation. In contrast, mopane woodland and mopane scrub generally occur on clay soils, prone to seasonal flooding (Smith 1998) and difficult to excavate in the dry season. Warthog have been reported to thrive in areas of wooded grassland bounding suitable floodplain grassland (Rodgers 1984), a situation which occurs especially towards the south of the transect study area in LNP. The close proximity of patches of aquatic grassland to burrows makes suitable dry season grazing readily available. The close ecological association between warthog and tsetse was outlined earlier in the Introduction including the observation that apparent densities of *G. m. morsitans* tsetse are greatest in *Combretum* woodland and *G. pallidipes* in thicket (Anderson 2009). It is very notable, therefore, that the majority of warthog burrows are located within these two habitats.

### Habitat Densities

The use of the classified dataset also allowed the aggregate density of wild mammals to be examined by vegetation class. Not surprisingly, the highest densities were recorded in grassland with nutritious herbage providing for large densities of puku in particular. Although lower densities of large mammals were recorded in the riverine woodland and aquatic grassland classes, these habitats are likely to be very important ecologically, especially in the dry season as a source of forage and water. They may support a wide diversity of other species not included in the survey such as birds, amphibians and invertebrates. Acacia woodland forms only a very small component of the vegetation in LNP and animals were rarely observed in the dense stands of *Acacia kirkii*, but were more commonly seen in more open acacia woodland near the Luangwa River.

It would have been desirable to estimate individual species density by habitat type, but the data were not robust enough to allow this. The large survey effort required (approximately 60 observations per habitat type for each species) makes this difficult to achieve across all habitat types, especially in environments with low mammal densities. Similarly, four land cover classes (riverine woodland, thicket, mopane scrub and *Combretum* woodland) did not have sufficient observations to enable the use of the stratum detection function in the analysis. Although preferable to using the global detection function, an accurate estimate was made possible by pooling the class in question with the class with the most similar visibility characteristics and using the global detection function for the two classes combined to estimate density. Riverine woodland was pooled with thicket for this purpose, and mopane scrub was pooled with *Combretum* woodland. Although species and cluster size may have confounded the detection probabilities to some degree, the estimated densities provide a useful indication of the general distribution of mammals.

### Size of the Park

Calculation of the area of LNP using the classified image (331 km^2^) produces a considerably different value to the official figure for the park area (254 km^2^). Unfortunately, it is not clear where the official figure used by the ZAWA originates from. Changes in the course of the Luangwa River forming the western boundary of the park are occurring continuously, but will not account for such a large discrepancy. Although the exact boundary of the park is disputed by the local community, the shapefile used in this study was created through digitisation of high resolution topographical maps (Surveyor-General 1972) based on the original gazetting of the park, which suggests the national park area figures used by ZAWA may be incorrect.

## Conclusion

This study provides a reliable framework for ecological monitoring of vegetation composition and wild mammal densities in remote, relatively inaccessible environments. Information generated can be used as a baseline for further study into wildlife disease systems. The use of classification algorithms based on fuzzy set theory enables accurate classification of vegetation classes despite the presence of natural mosaics and mixed pixels. The datasets created are ideal for use as a GIS base layer for the design, implementation and analysis of ecological and epidemiological studies. The distance sampling technique utilising a ground survey allows for reliable estimation of densities of smaller mammal species and important hosts of trypanosomes such as warthogs. The large survey effort required to estimate species density accurately in areas with relatively low wild mammal densities may limit the usefulness of this technique for health research in some environments.

Despite decades of research into trypanosomiasis our understanding of disease transmission in wildlife hosts is limited by the complexity and large size of the reservoir host community, and the many factors that influence it. Accurate description of the structure and distribution of communities is necessary to further our understanding and will enable better management of health relationships in remote environments such as those described in this study. Data such as these will help to enable improved modelling of disease systems with a consequential improvement in our understanding of the effects of interventions in biodiverse ecosystems.

## Footnotes

1 Physiognomic refers to the overall structure or physical appearance of the community including appearance, height and spacing.
